# Educational interventions to improve bowel cancer awareness and screening in Organisation for Economic Co-operation and Development countries: A scoping review

**DOI:** 10.1016/j.pmedr.2024.102653

**Published:** 2024-02-13

**Authors:** Nicola Gadd, Simone Lee, Matthew J Sharman, Kehinde Obamiro

**Affiliations:** a Centre for Rural Health, School of Health Sciences, University of Tasmania, Launceston 7250, Australia; b School of Health Sciences, University of Tasmania, Launceston 7250, Australia; c Central Queensland Centre for Rural and Remote Health, James Cook University, Emerald, Queensland, Australia

**Keywords:** Bowel cancer, Bowel cancer screening, Awareness, Knowledge, Education intervention, Counselling

## Abstract

**Objectives:**

Summarise theory informed educational interventions for improving bowel cancer awareness and screening.

**Methods:**

A search was conducted in PubMed, EMBASE, Web of Science and CINAHL. English studies from 2016 to 2022 which implemented community-based bowel cancer awareness and/or screening education interventions for adults in Organisation for Economic Co-operation and Development countries were included.

**Results:**

Sixty-two studies were included, 32 measured both screening and awareness (24 measured screening only, 6 measured awareness only). Education interventions were grouped and summarised in five education types: lay community health education/counselling (n = 28), education material (n = 5), health professional education/counselling (n = 10), mass media (n = 5) and other (n = 19). Other included education interventions which did not fit into the four types previously mentioned. Six studies tested more than one education type. Each type within these studies were reported/summarised separately within the appropriate education type. Lay educators resulted in improved awareness and screening. Brochures were effective education materials for screening and combined with lay educators resulted in increased awareness. State-wide mass media campaigns significantly improved screening uptake for up to 2-months post-campaign. Fear and loss-framed messaging improved screening intentions compared to humour or gain-based messaging. Decision aids had limited improvements in awareness. Facebook campaign and telephone counselling had limited improvements in screening.

**Conclusions:**

Lay community health educators, brochures, and mass media campaigns occurring multiple times a year may be effective interventions in improving screening and/or awareness. Such approaches should be considered when developing community education. Education interventions should include multiple components suggested above to maximise improvements of awareness and screening.

## Highlights

• Main types of education interventions included lay community health educators, health professional educators/counselling, education materials, mass media campaigns and other types. The ‘other’ types included 12 different education intervention strategies which did not fit into the four types above, for example, a theatre play, women’s health day, a video, decision aid, or Facebook group or promotion.
• Lay community health educator education showed improvements in both screening participation and awareness of bowel cancer and screening.
• Frequent state-wide mass media campaigns run throughout the year can increase screening participation by encouraging new participants or re-screeners when they become eligible.
• Facebook campaigns and telephone counselling had limited improvements in screening participation.

## 1 Introduction

Bowel cancer is the third most common cancer and the second highest leading cause of cancer deaths globally (Global Colon Cancer Association, 2021b). Research suggests it is linked to lifestyle factors, including physical inactivity, poor diet, tobacco smoking and high alcohol consumption (Rawla et al., 2019, World Cancer Research Fund & American Institute for Cancer Research, 2018, Cancer Research UK. What is bowel cancer Updated, 2021). To tackle this problem, it is recommended to raise awareness of the following about bowel cancer: incidence rates, prevention, and how early detection can lead to successful treatment (Global Colon Cancer Association, 2021b). Research has shown that bowel cancer is most preventable and highly treatable when detected early, with a five-year survival rate of >90 % compared to 13 % when detected at a later stage (m^2^). Screening has contributed to reduced mortality rates globally by ensuring detection and subsequent removal of pre-cancerous polyps (Rawla et al., 2019). There is evidence to suggest that >50 % reduction in bowel cancer mortality rates between 1975 and 2010 in USA can be attributed to screening (Zauber, 2015).

Many Organisation for Economic Co-operation and Development (OECD) countries have implemented nation-wide bowel cancer screening programs including Australia, France, United Kingdom, New Zealand, Ireland, and Germany (Martini et al., 2016). Most of these programs are implemented through home test kits sent via postal mail to eligible individuals aged >50 years (Global Colon Cancer Association, 2021a, Australian Government Department of Health, 2022, Health Service Executive. Bowel screening - BowelScreen. Updated, 2019, New Zealand Government Ministry of Health, n.d., National Health Service. Overview bowel cancer Updated, 2021). These test kits include inexpensive stool tests via the faecal occult blood test (FOBT) or faecal immunochemical test (FIT) (Global Colon Cancer Association, 2021a). Since implementing these screening programs, participation rates have been low for many of these countries (Global Colon Cancer Association, 2021a). Current data suggests only 40.9 % (2020–21) of the eligible population participate in the national program screening in Australia, and 46.6 % in Ireland (2020–21) (Australian Institute of Health and Welfare, 2023, Health Service executive. BowelScreen Programme Report, 2022). Higher participation rates have been observed in England in recent years with up to 69.6 % in 2021–22 (Cancer Research UK. Bowel Screening Uptake. Updated, 2023). Strategies to improve screening participation are therefore needed to allow these programs to achieve their objectives. An Australia study showed an increase in participation to 60 % could prevent a further 37,300 cases and 24,800 deaths (Lew et al., 2017). Raising awareness of risk factors, symptoms and early detection through screening is one way to achieve this. Previous studies have implemented different types of interventions to increase bowel cancer awareness and screening participation. One study implemented an education intervention through a community pharmacy awareness program (Sendall et al., 2018). Another study implemented two state-wide interventions and compared the impact on screening participation (Lofti-Jam et al., 2019). A state-wide television mass media campaign promoting the National Bowel Cancer Screening Program in Australia was also compared with a lower-intensity promotion method of a television advertisement, printed and online advertising (Lofti-Jam et al., 2019). Understanding the study designs, how the interventions were implemented and whether the interventions were successful may assist in the development of successful education interventions to improve awareness and screening participation.

As most OECD countries continue to have low screening rates, increasing public awareness of symptoms and risk factors may encourage improved screening participation (Kanavos and Schurer, 2010). Several reviews have been conducted previously, including, reviews of bowel cancer screening-only interventions in clinical settings (Schliemann et al., 2021, Dougherty et al., 2018), a review of small media influencing FOBT screening (Baron et al., 2008) and a 2016 review of community-based promotion interventions to improve awareness and screening (Martini et al., 2016). As the 2016 review excluded interventions based on behavioural change models (Martini et al., 2016), the current review aimed to update and broaden this evidence by summarising community education interventions (including theory-informed) for improving bowel cancer awareness and screening in OECD countries.

## 2 Methods

This scoping review was conducted according to the JBI Manual for Evidence Synthesis (Peters et al., 2020) and reported according to PRISMA-ScR (Preferred Reporting Items for Systematic Reviews and Meta-analyses extension for Scoping Reviews) checklist (Tricco et al., 2018). This review was conducted in accordance with methodology described by Arksey and O’Malley (Arksey and O’Malley, 2005) and Colquhoun et al. (Colquhoun et al., 2014). See Table 1 for the eligibility criteria.

**Table 1:** Inclusion and exclusion criteria for the studies in the scoping review.

	Inclusion	Exclusion
Year	2016–2022 (updated following preliminary searches in the four databases)	2015 and prior; 2023 and later
Language	English	All other languages
Countries	OECD countries	All other countries
Population	>18 years General population	<18 years Health care professionals High-risk population groups for bowel cancer
Article type	All study designs which reported the results of the interventions. Grey literature from identified peer-reviewed literature reference lists.	Review papers Conference abstracts Articles which did not implement an intervention
Concept	Bowel cancer awareness and screening education interventions in community settings	Bowel cancer awareness and screening education interventions in clinical settings Education for bowel preparation for colonoscopy Patient reminders

Primary outcome measures were bowel cancer awareness and screening levels following educational interventions, focusing on the interventions design, implementation, and findings. Awareness outcomes were measured through awareness and knowledge and screening through uptake or intentions.

The search was conducted using four databases, PubMed, EMBASE, Web of Science and CINAHL. Search strategies were altered to fit the database's search capacities in consultation with a research librarian (Appendix A). After the preliminary searches retrieved 5692 studies, the eligibility criteria were updated, and studies were limited to English studies published from 2016 to 2022. The 2016 limit was agreed upon due to a similar review conducted, to synthesise evidence from contemporary studies (Martini et al., 2016). Database searches had weekly alerts to identify relevant studies to include until September 2022. Grey literature was searched by checking included studies reference lists.

**Fig. 1:**
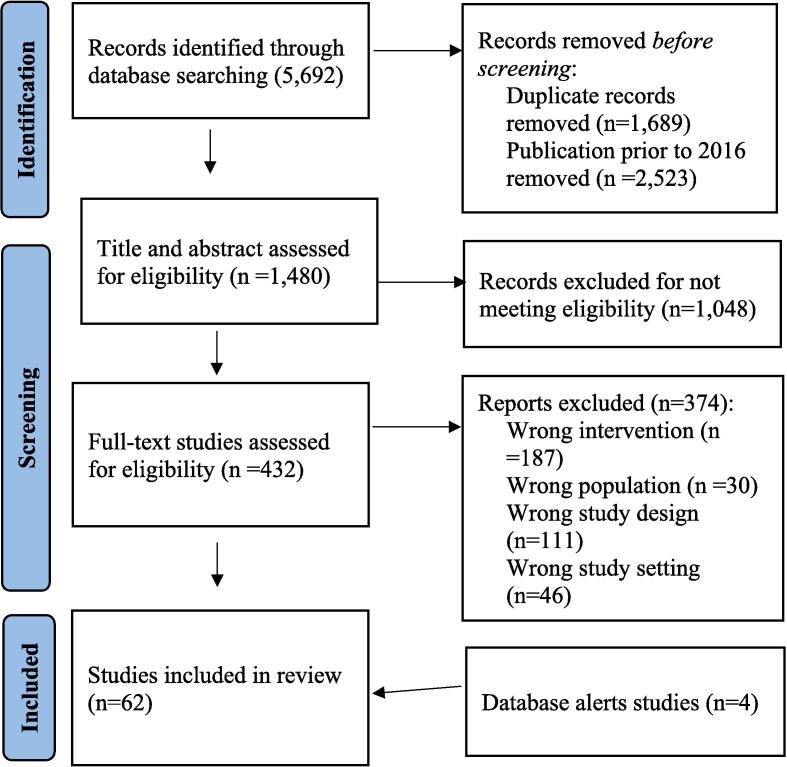
PRISMA flow diagram of the search strategy followed for the scoping review.

Database search results were exported into EndNote software (Clarivate, 2022). One researcher (NG) screened references in EndNote to remove duplicate, non-OECD country and studies prior to 2016. Covidence software (Innovation VH, n.d) was used to screen studies. Title and abstract and full-text screening was conducted by two researchers independently. Disputed studies were resolved through consensus or a third researcher’s input. Search results are displayed on a PRISMA diagram (Fig. 1) (Page et al., 2021). Data was extracted from included studies using a data extraction template in Covidence software (Veritas Health Innovation, n.d). The authors adapted the template from the JBI Manual for Evidence Synthesis (Peters et al., 2020) (Appendix B) and piloted it using two studies (Shepherd et al., 2022, Fernandez et al., 2022). Data was extracted by one author (NG) and checked by another (SL, KO, MS). Data extracted from included studies were categorised into themes based on education intervention types to compare results within and between themes.

## 3 Results

**Fig. 2:**
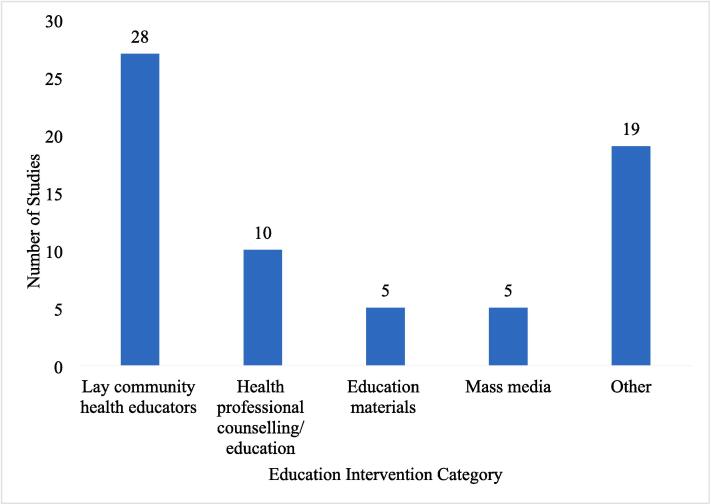
The types and the number of bowel cancer awareness and screening interventions used among the included studies in the scoping review.

The search retrieved 1480 studies for screening (Fig. 1). Title and abstract screening removed 1048 from unrelated topics to bowel cancer awareness and screening educational interventions. Full text screening removed 374; 58 studies remained. Reference list checks did not retrieve additional studies or grey literature. Databases alerts retrieved 4 additional studies, resulting in 62 included studies. The inter-rate agreement (Cohen’s Kappa) between authors title and abstract screening were NG, KO: 0.53; NG, MS: 0.67; NG, SL: 0.48. NG screened all papers and KO, MS, and SL shared screening equally. Countries included USA (n = 48, 77.4 %), United Kingdom (n = 4, 6.4 %), Australia, France, Netherlands, Canada (n = 2, 3.2 % each), Denmark and Switzerland (n = 1, 1.6 % each). Study designs were randomised-control trials (n = 27, 45.1 %), non-randomised experimental trials (n = 18, 29.0 %), cross-sectional studies (n = 12, 19.3 %), cohort studies (n = 3, 4.8 %), mixed methods (n = 1, 1.6 %), and a case report (n = 1, 1.6 %). Screening uptake or intentions were reported in 54 studies (n = 20 intentions, n = 33 uptake, n = 1 both), and 33 studies reported knowledge or awareness outcomes (n = 27 knowledge, n = 5 awareness, n = 1 both). Types of interventions included lay community health educator education/counselling (LCHEE), (n = 28), health professional education/counselling (HPE) (n = 10), education materials (n = 5), mass media (n = 5) and other types (n = 19) (Appendix C, Fig. 2, see definitions of types in Table 2). Some studies reported on several education types.

**Table 2:** Definitions of the types of education interventions identified in the scoping review and the key findings of each type.

Intervention Type	Definition	Key Findings
Lay community health educator education/counselling (LCHEE) (n = 28)	Education sessions or counselling conducted by a lay community health educator (a non-health professional).	**Awareness: (n = 22)** - All four guided inflatable colon tours improved awareness post-tour but varied in the levels of increase (32–35).- Church counselling increased knowledge for all three studies although, only one reported statistical significance (36–38). Another study provided faith-based motivational interviewing and found moderate knowledge improvements (39).- Culturally and low literacy tailored LCHEE significantly improved knowledge compared to the control group (40) . - LCHEE in a workplace setting significantly improved knowledge (41).- LCHEE and social support significantly improved knowledge (42) .- Peer-led LCHEE positively changed awareness although statical significance was not reported (43) . **Screening: (n = 26)**- Social support assisted with an increase in screening uptake in six studies (41, 42, 44–47). Of those studies, three tested statistical significance with two significant results (42, 47).- Community outreach events had improved screening uptake (48) .- Providing screening kits (FITs) directly to individuals improved screening uptake (42, 44, 49) . - Inflatable colons did not significantly improve screening intentions; two studies found no change (32, 34), one found no difference between groups (35) and one found a small increase (35).- Church LCHEE did not significantly improve screening uptake among five studies (36–38, 46, 50). Two of those studies found no statistically significant difference in screening uptake between LCHEE and control groups (46, 50).- LCHEE with over three education sessions increased screening uptake compared to one session in two studies (36, 49) . **Awareness and screening:**- Phone counselling was more effective than an interactive computer program for both awareness and screening uptake (51) .- Lay educator education and patient navigation improved awareness but not screening uptake (52) .- Child lay educators educating their families were successful for both awareness and screening intentions (53) .
Health professional education/counselling (HPE) (n = 10)	Education sessions or counselling conducted by a health professional.	**Awareness: (n = 6)** - Two studies (a home health party and pharmacist counselling) measured change in awareness baseline and post-education with statistically significant increase in awareness. Although, no difference was observed between the pharmacist counselling and control group (54, 55).- Two studies only reported on the ease to understand the information provided or learning something new oppose to measuring a change in awareness/knowledge (56, 57) . **Screening: (n = 9)**- The pharmacy campaign and counselling improved screening uptake (55, 58) .- Nurse/psychologist counselling was more effective at increasing screening intentions among those who were first time screeners or had screened previously. There was no change in intentions for those who refused to screen (59) .- Home health parties significantly improved screening uptake (54) .- Physician and LCHEE sessions improved screening intentions and uptake among participants with culturally barriers to handling stools (57) . **Awareness and screening:**- A mobile bus clinic approach with physician and nurse-led education was able to educate 772 community members about bowel cancer signs and symptoms. The level of awareness was not measured (60) . This approach was also able to reduce hospital wait times by 4.6 weeks for screening by performing 244 sigmoidoscopies on the bus.
Education materials (n = 5)	Written education materials (brochures, instructions) used for assisting education only.	**Awareness: (n = 5)**- Brochures plus lay educators combined were significantly more effective than brochures alone for awareness for two of three studies (61–63).- Brochures led to higher awareness than photonovella (64) . **Screening: (n = 4)** - All three studies comparing brochures with brochures plus LCHEE found similar screening increases between groups (61–63). - Only one study found a significant increase in screening following brochures plus LCHEE and brochures alone (61).- Brochures plus LCHEE increased screening among those not up to date with screening. Brochures alone did not (62) .- Increased screening was observed for education brochures and photonovella although not statistically significant (63, 64) .
Mass media (n = 5)	Education methods conducted through media.	**Awareness: (n = 2)** - A campaign with television advertisements, billboards, and bus stop posters significantly increased knowledge of some but not all bowel cancer symptoms compared to the control group (65). **Screening: (n = 4)**- A Facebook campaign was less effective than the state-wide advertisements at improving screening uptake (66) .- State-wide screening campaigns increased screening for a limited time post-campaign (67, 68) .
Other (n = 19)	All other education interventions which do not fit in the other four types.	**Awareness: (n = 7)**- A culturally tailored video with two group workshops improved awareness (69) . **Screening: (n = 18)**- Text message with screening testimonials did not increase screening intentions compared to no text message (70) . - Higher screening intentions were observed among three studies through fear or loss-based messaging compared to humour or gain-based messaging about bowel cancer (66, 71, 72). **Awareness and screening:**- A women’s health day improved participants awareness and screening uptake/intentions (73) . - A women’s health day educated women on bowel health, screening and provided FOBTs for participants and their husbands.- A theatre play with a booth to book screening appointments found knowledge and screening intentions increased post-play (74) . - The lowest awareness improvements in ‘other’ were observed through three decision aid interventions, with no between group differences between the test and control groups (75–77). - Two of these, improved knowledge (75, 76) and one no change (77). - For screening, two studies improved screening intentions/uptake (75, 77) and one found no significant difference in screening intentions between the decision aid and control groups (76).

### 3.1 Education interventions to improve bowel cancer awareness/knowledge

#### 3.1.1 Education materials

Four studies reported findings of education materials to improve awareness (two education materials plus LCHEE (Jo et al., 2017, Nguyen et al., 2017); two education materials only (Christy et al., 2016, Fransen et al., 2017). All studies reported findings on brochures, one with photonovella and one with a national screening kit. Fransen et al. (Fransen et al., 2017) interviewed participants with low health literacy about the accessibility and comprehensibility of a national screening kit. Screening knowledge improved for 10 of 16 items measured (Appendix C), excluding information about risk, voluntary screening, and screening sensitivity (Fransen et al., 2017). Fransen et al. (Fransen et al., 2017) found low health literacy individuals may benefit from other methods (pictorial, animations, narratives) than a brochure and instructions to explain screening. Participants browsed the brochure for pictures, read headings, and reported there was too much information. Some information was confusing for example, the FOBT was not diagnostic, or the difference between FOBT and colonoscopy as the follow up diagnostic test (Fransen et al., 2017).

Eight studies used flipcharts to aid awareness and screening education (n = 7 both, n = 1 awareness) although, did not report findings on flipcharts (Molokwu et al., 2017, Tong et al., 2017, Mojica et al., 2016, Briant et al., 2018, Chow et al., 2020, Cassel et al., 2020, Jo et al., 2017, Cuaresma et al., 2018). Similarly, nine studies used brochures to aid education (n = 1 screening, n = 1 awareness, n = 7 both) although, only five reported brochure-specific findings (findings discussed in this section and below) (Dominic et al., 2020, Woodruff et al., 2017, Naguib et al., 2017, Jo et al., 2017, Nguyen et al., 2017, Cuaresma et al., 2018, Christy et al., 2016, Fransen et al., 2017, Mukherjea et al., 2020).

#### 3.1.2 Lay community health educator education or counselling

Twenty-two studies used LCHEE for awareness interventions (Boutsicaris et al., 2021, Miguel et al., 2020, Portilla-Skerrett et al., 2019, Molina et al., 2018, Holt et al., 2019, Maxwell et al., 2019, Maxwell et al., 2020, Jenkins et al., 2022, Molokwu et al., 2017, Warner et al., 2019, Dominic et al., 2020, Gray et al., 2021, Ou et al., 2019, Tong et al., 2017, Woodruff et al., 2017, Mojica et al., 2016, Parker et al., 2021, Cassel et al., 2020, Jo et al., 2017, Nguyen et al., 2017, Cuaresma et al., 2018, Lucas et al., 2021). Nineteen studies showed improvements although, the findings of three were not statistically significant. Woodruff et al. (Woodruff et al., 2017) focused on 23 community outreach events; 74 % of participants correctly identified their screening status post-events. Christy et al. (Christy et al., 2020) compared web-based program with phone counselling; phone counselling increased knowledge more. Eight other studies conducted LCHEE: seven reported knowledge/awareness increases. Of those, four found a knowledge increase, three were statistically significant (Warner et al., 2019, Dominic et al., 2020, Mojica et al., 2016), one was not (Gray et al., 2021). Three reported higher knowledge increases for the intervention groups compared to control, one was statically significant (Molokwu et al., 2017), two were not (Tong et al., 2017, Cuaresma et al., 2018).

#### 3.1.3 Health professional education or counselling

Five studies had HPE for awareness interventions; a physician-led presentation (n = 1) (Hoffman et al., 2016), physician and nurse-led education (n = 1) (Chow et al., 2020), pharmacist-led counselling (n = 1) (Holle et al., 2020), physician and LCHEE-led presentation (n = 1) (Cassel et al., 2020) and community health workers (n = 1) (Briant et al., 2018). Three studies measured change in awareness (Briant et al., 2018, Holle et al., 2020, Hoffman et al., 2016). Hoffman et al. (Hoffman et al., 2016) found mixed results in awareness changes post-physician-led presentation. Chow et al. (Chow et al., 2020) provided counselling to rural individuals staying at a city-based lodge awaiting healthcare; 98 % felt they were provided sufficient information about screening (Chow et al., 2020). The physician and LCHEE-led presentation had 92 % participants report they learnt something about bowel health (Cassel et al., 2020).

#### 3.1.4 Mass media

Two studies used mass media for awareness education (Torrance et al., 2021, Katz et al., 2017). Katz et al. (Katz et al., 2017) found billboards and posters easy to understand the message and few participants saw the newspaper articles making them less effective.

#### 3.1.5 Other

Seven studies used other types of educational interventions for awareness education: a video (n = 1) (Nakajima et al., 2022), Facebook group (n = 1) (Key et al., 2020), decision aid (n = 3) (Gabel et al., 2020, Woudstra et al., 2019, Housten et al., 2020), theatre play (n = 1) (Friedman et al., 2019), and a women’s health day (n = 1) (McBride and Gesink, 2018). The theatre play, Women’s health day and culturally tailored video with two group workshops all displayed the most improvements. The study found 73 % participants felt they understood the content covered (McBride and Gesink, 2018). Facebook group intervention participants reported learning how to decrease their risk (Key et al., 2020).

### 3.2 Education interventions to improve bowel cancer screening

#### 3.2.1 Education materials

Four studies reported findings of education materials to improve screening (three education material and LCHEE (Jo et al., 2017, Nguyen et al., 2017, Cuaresma et al., 2018), one education material only (Christy et al., 2016). Christy et al. (Christy et al., 2016) found 86.7 % participants completed screening (82 % photonovella group: 90 % brochure group). See Table 2 and Appendix C for further findings.

##### 3.2.1.1 Lay community health educator education or counselling

Twenty-five studies used LCHEE for screening education (Boutsicaris et al., 2021, Miguel et al., 2020, Portilla-Skerrett et al., 2019, Molina et al., 2018, Holt et al., 2019, Maxwell et al., 2019, Maxwell et al., 2020, Warner et al., 2019, Dominic et al., 2020, Gray et al., 2021, Ou et al., 2019, Rafie et al., 2020, Elder et al., 2017, Tong et al., 2017, Woodruff et al., 2017, Maxwell et al., 2016, Leone et al., 2016, Christy et al., 2020, Mojica et al., 2016, Parker et al., 2021, Jo et al., 2017, Nguyen et al., 2017, Cuaresma et al., 2018, Sizer and Conyers, 2022, Champion et al., 2018). Ten studies reported screening intentions (Boutsicaris et al., 2021, Miguel et al., 2020, Portilla-Skerrett et al., 2019, Molina et al., 2018, Maxwell et al., 2019, Warner et al., 2019, Dominic et al., 2020, Gray et al., 2021, Mojica et al., 2016, Parker et al., 2021). Six of those reported improvements (Miguel et al., 2020, Molina et al., 2018, Warner et al., 2019, Woodruff et al., 2017, Gray et al., 2021, Parker et al., 2021) and three reported no change (Boutsicaris et al., 2021, Portilla-Skerrett et al., 2019, Mojica et al., 2016). Mojica et al. (Mojica et al., 2016) found no difference between those who did and did not attend the education. Parker et al. (Parker et al., 2021) had children teach their families, 100 % of families increased screening intentions post-event. Dominic et al. (Dominic et al., 2020) provided social support from loved ones to support screening and found significant improvements. Social support group were 2.1700D7 more likely to screen then the control; 66 % and 47.2 % completed FITs respectively (Dominic et al., 2020).

Fourteen studies reported screening uptake (Holt et al., 2019, Maxwell et al., 2020, Ou et al., 2019, Rafie et al., 2020, Elder et al., 2017, Tong et al., 2017, Woodruff et al., 2017, Maxwell et al., 2016, Leone et al., 2016, Christy et al., 2020, Jo et al., 2017, Nguyen et al., 2017, Cuaresma et al., 2018, Sizer and Conyers, 2022). Improvements varied; 26 % increase (Ou et al., 2019), 20.6 % increase (Rafie et al., 2020), and Cuaresma et al. (Cuaresma et al., 2018) had 9 % increase (intervention); 1 % increase (control) although, not statistically significant. Sizer et al. (Sizer and Conyers, 2022) trained a barber to provide LCHEE for clients, 70 % booked a colonoscopy post-intervention. Maxwell et al. (Maxwell et al., 2016) found enhanced education had 83 % more participants screen compared to basic education.

##### 3.2.1.2 Health professional education or counselling

Nine studies used HPE for screening education; physician and nurse-led education (n = 2) (Chow et al., 2020, Naguib et al., 2017), pharmacist-led counselling (n = 2) (Holle et al., 2020, Ruggli et al., 2019), physician-led presentation (n = 1) (Mukherjea et al., 2020), nurse practitioner and clinic staff education (n = 1) (O’Keefe et al., 2018), community health worker (n = 1) (Briant et al., 2018), nurse and psychologist counselling (n = 1) (Denis et al., 2017), and a physician and LCHEE-led presentation (n = 1) (Cassel et al., 2020). The physician and nurse-led bus clinic screened 32 % of participants, 92 % reported they would use the bus again (Naguib et al., 2017). The other physician and nurse study reported 32 % participants were provided FOBTs (Chow et al., 2020). Ruggli et al. (Ruggli et al., 2019) found 47 % participants would not have screened without the pharmacist campaign. The physician-led presentation improved screening intentions among non-screeners but were higher among those who had screened previously (Mukherjea et al., 2020). The nurse practitioner employee wellness program had on-site screening kits provided to employees where they could pick up and drop off kits. Both study sites had >70 % participants complete FITs provided (35 % screening increase) (O’Keefe et al., 2018). The nurse and psychologist counselling study found no between group differences for screening intentions (Denis et al., 2017).

##### 3.2.1.3 Mass media

Four studies used mass media for screening education, via public awareness campaigns (n = 3) (Durkin et al., 2020, Katz et al., 2017, Durkin et al., 2019b) and a Facebook campaign (n = 1) (Koivogui et al., 2020). Two public awareness campaign studies reported population change in screening rates, with higher rates for campaign compared to non-campaign states during campaign weeks. Screening increases were limited; up to one-month (Durkin et al., 2020) and 2-months (Durkin et al., 2019b) post-campaign. Katz et al. (Katz et al., 2017) compared screening intentions between campaign and control groups, with no significant differences observed. The Facebook campaign had limited reach of 8.9 % of target population, only 0.75 % requested a kit and 0.16 % completed screening (Koivogui et al., 2020).

##### 3.2.1.4 Other

Eighteen studies used other educational interventions for screening education; a video (n = 2) (Carcioppolo et al., 2020, Lucas et al., 2021), Facebook promotion (n = 1) (Lee-Won et al., 2017), audio (n = 1) (Kennedy et al., 2018), online education (n = 2) (Lucas et al., 2016, Lucas et al., 2018), Facebook group (n = 1) (Key et al., 2020), decision aid (n = 3) (Gabel et al., 2020, Woudstra et al., 2019, Housten et al., 2020), theatre play (n = 1) (Friedman et al., 2019), women’s health day (n = 1) (McBride and Gesink, 2018), text message (n = 1) (Alber and Glanz, 2018), online message (n = 2) (Champion et al., 2018, Neil et al., 2022), patient navigation (n = 1) (Fernandez et al., 2022), and a newsletter (n = 2) (Shepherd et al., 2022, Leone et al., 2016). Five studies measured screening uptake (Shepherd et al., 2022, Leone et al., 2016, Gabel et al., 2020, Key et al., 2020, Champion et al., 2018). The Facebook group found no change in screening (Key et al., 2020) and one newsletter study found no statistically significant difference between the control and newsletter groups (Leone et al., 2016). Another newsletter study found 3.9 % of those sent the newsletter engaged in study (Shepherd et al., 2022). Of those, 64.5 % completed screening. An online message study (Neil et al., 2022) and text message study (Alber and Glanz, 2018) both observed no significant differences in screening intentions between groups.

Varied intervention formats compared the framing of messages to improve screening intentions or uptake including video (n = 2) (Carcioppolo et al., 2020, Lucas et al., 2021), Facebook promotion (n = 1) (Lee-Won et al., 2017), and an online education module (n = 2) (Lucas et al., 2016, Lucas et al., 2018). Loss-framed messaging consistently improved screening uptake or intentions in five studies (Carcioppolo et al., 2020, Lee-Won et al., 2017, Lucas et al., 2021, Lucas et al., 2016, Lucas et al., 2018) and was more effective compared to gain-framed messaging. Facebook promotion study (Lee-Won et al., 2017) and a video study (Carcioppolo et al., 2020) both found loss-framed messaging was associated with colonoscopy intentions through inducing fear. Lee-Won et al. (Lee-Won et al., 2017) reported fear-evoking messages may encourage screening by highlighting the harms to one’s health if they do not screen. Both studies suggested loss-framed messages were more useful for individuals without bowel cancer worry by inducing emotions (Carcioppolo et al., 2020, Lee-Won et al., 2017). Those with worries may benefit from humour-framed messages (Carcioppolo et al., 2020). Message framing studies found culturally tailored messaging had more improvements than standard-messaging among African American participants (Lucas et al., 2021, Lucas et al., 2018). Lucas et al. (Lucas et al., 2018) reported those with higher racial identity benefited more with additional culturally tailored message.

#### 3.2.2 Summary of evidence for bowel cancer awareness/knowledge and screening education

Child LCHEE (Parker et al., 2021), a home health party (Briant et al., 2018), a women’s health day (McBride and Gesink, 2018) and a local theatre play (Friedman et al., 2019) all improved awareness and screening intentions and should be further explored. LCHEE faith-based education and counselling is an option to explore further for improving awareness/knowledge as all four studies showed improvements, two reported statistically significance (Appendix C) (Holt et al., 2019, Maxwell et al., 2019, Maxwell et al., 2020, Jenkins et al., 2022). Similarly with inflatable colon tours (Boutsicaris et al., 2021, Miguel et al., 2020, Portilla-Skerrett et al., 2019, Molina et al., 2018). Although, both LCHEE faith-based education and the inflatable colon tour approaches did not show promising results for improving screening uptake and/or intentions. Reasons for this may be screening barriers such as income, health insurance and/or fear of the screening procedure or cancer diagnosis (Boutsicaris et al., 2021, Portilla-Skerrett et al., 2019). Portilla-Skerrett (Portilla-Skerrett et al., 2019) found participants reported fear of cancer diagnosis (44 %) and screening procedure (14 %) as factors not to screen. Boutsicaris (Boutsicaris et al., 2021) reported knowledge alone was not enough to change the screening behaviour, as the barriers of health insurance and income had statically significant associations with screen intent. All the inflatable colon tour and faith-based education studies were conducted in USA where cost and health insurance can be barriers to screening. These two LCHEE options should be explored in countries with free screening options to measure the impact on screening intent/uptake with such barriers removed.

Considering the evidence for awareness/knowledge, it appears that LCHEE approaches, brochures plus LCHEE or a culturally tailored video and workshops approaches may be best for improving bowel cancer awareness. Nineteen (86.4 %) LCHEE studies reported improvements in awareness. Brochures plus LCHEE improved awareness more than education materials alone (Jo et al., 2017, Nguyen et al., 2017, Cuaresma et al., 2018). As for screening, both brochures and brochures plus LCHEE were effective although, only brochures plus LCHEE increased screening among those not up-to-date (Jo et al., 2017, Nguyen et al., 2017, Cuaresma et al., 2018). A culturally tailored video with two group workshops improved awareness but did not measure screening (Nakajima et al., 2022).

Considering the evidence for screening, it appears LCHEE with social support, directly providing screening, brochures plus LCHEE, statewide mass media campaigns, some health professional approaches and fear or loss-framed messaging were most effective for screening uptake and/or intentions. LCHEE and social support were effective at improving screening uptake among six studies (Warner et al., 2019, Dominic et al., 2020, Ou et al., 2019, Rafie et al., 2020, Elder et al., 2017, Tong et al., 2017). Directly providing screening options to participants may also encourage uptake (Boutsicaris et al., 2021, Dominic et al., 2020, Ou et al., 2019, Maxwell et al., 2016, Ruggli et al., 2019, Christy et al., 2016, McBride and Gesink, 2018, O’Keefe et al., 2018). This was observed among several education types. Statewide mass media campaigns were effective at improving screening uptake in the short-term (up to 2-months post campaign) (Durkin et al., 2020, Durkin et al., 2019a).

Evidence measuring awareness is lacking for mass media campaigns. Health professional approaches pharmacist counselling campaigns (Holle et al., 2020, Ruggli et al., 2019) and mobile bus clinic (Naguib et al., 2017) reached large participants numbers for education. These approaches showed promising results for screening uptake but reported limited or no findings on awareness which should be further explored. Physician and LCHEE sessions were able to overcome cultural barriers and improve screening intentions and uptake (Cassel et al., 2020). Such approach should be further explored. Lastly, fear or loss-based messaging showed more favourably than humour or gain-based messaging at improving screening intentions and should be considered in education approaches (Carcioppolo et al., 2020, Lee-Won et al., 2017, Lucas et al., 2021).

## 4 Discussion

The present review broadened evidence from a 2016 review of 18 studies on bowel cancer awareness and screening promotional campaigns (Martini et al., 2016). The 2016 review identified mass and small media, group and one-on-one education, financial support, special events and celebrity endorsements (Martini et al., 2016). All strategies except small media directly measured screening. Group education was identified as more effective than one-on-one education and financial support for screening. Only one mass media study measured knowledge, with an unspecified increase reported (Martini et al., 2016). The present review expanded on this including more studies measuring awareness/knowledge, and education specific interventions including those informed by behavioural change theories, and compared interventions by educators, health professionals or lay educators. This allowed the authors to summarise types of educators who could benefit specific groups for both awareness and screening. This review suggested several types of education: education materials, mass media, HPE, LCHEE and other. Some studies used combined education types; LCHEE were most common to improve awareness and screening. Studies used different methods for reporting and measuring results. Each education type broadly led to differences in outcomes.

### 4.1 Mass media

Awareness education was implemented through mass media campaigns in two studies, both led to improvements. Comparisons between these studies were difficult as awareness measures differed. One measured percentage increase in awareness (Torrance et al., 2021), the other reported whether participants found the campaign easy to understand (Katz et al., 2017). Four studies focused on mass media campaigns to improve screening and reported mixed results. Two showed improvements for up to 2-months post-campaign (Durkin et al., 2020, Durkin et al., 2019b). A study that used billboards, posters, and newspaper articles found no difference between campaign and control groups (Katz et al., 2017). Another used a Facebook campaign with minimal reach and screening uptake (Koivogui et al., 2020). These findings suggest billboard and Facebook campaigns may not effectively encourage large scale screening. Katz et al. (Katz et al., 2017) billboard campaign did not include television, radio, or internet advertising and Koivogui et al. (Koivogui et al., 2020) suggested Facebook campaigns could be useful for younger audiences (50–54 years) who had not screened previously. Suggesting both campaigns have lesser reach and screening uptake compared to state-wide campaigns with television, online, social media and radio advertisements.

State-wide campaigns may be more effective for screening although with short-term effects. Durkin et al. (Durkin et al., 2020) suggested first-time or never previously participated screeners were encouraged due to the high-reach media approach. Television advertisements were useful to target older individuals thus, combining high and lower-reach media advertisements could target different eligible age groups. Durkin et al. (Durkin et al., 2020) suggested campaigns should run throughout the year to encourage eligible individuals to screen and maximise effect. Similarly, the 2016 review reported improvements in screening for three mass media studies and reported an association between screening rates and frequency of exposure to campaigns (Martini et al., 2016).

### 4.2 Education material

Brochures were the most common education material. Flipcharts aided education sessions although lacked specific findings. Education materials improved both awareness and screening. Our finding’s showed brochures were more effective than photonovella to improve awareness and screening (Christy et al., 2016). Although awareness and screening findings differed for brochures compared to brochures plus LCHEE. For awareness, brochures were less effective than brochures plus LCHEE (Nguyen et al., 2017, Cuaresma et al., 2018). Perhaps due to the relationship between participants and educators and the repetition of information provided through varied modes of providing information, for example, having written brochures and verbal communication through LCHEE (Nguyen et al., 2017). In contrast, both interventions showed similar improvements in screening. Suggesting the addition of LCHEE may not encourage more screening participation compared to brochures alone. Though, Jo et al. (Jo et al., 2017) proposed brochures may have been sufficient to educate participants to screen without the need for further LCHEE, as participants had high education levels. Similarly, in Cuaresma (Cuaresma et al., 2018), which found no significant difference between groups, showed the control group had higher education levels compared to the intervention group. Thus, lower educated individuals may benefit from both for screening. Therefore, for most improvements in awareness and whole population approaches (low and high education levels) for screening, education interventions could combine brochures with LCHEE.

Findings showed providing a brochure with screening kit instructions may improve awareness (Fransen et al., 2017) although, the effectiveness of education materials may vary depending on individuals’ health literacy levels. Those with low health literacy are less likely to complete stool tests from higher perceived barriers for example, it is embarrassing, confusing, and difficult (Arnold et al., 2012). Coronado et al. (Coronado et al., 2014) compared wordless (low health literacy) FIT instructions with worded instructions, to compare the understandability and acceptability. Participants preferred wordless instructions, with higher understandability and more user-friendly for low health literacy or non-English speaking individuals (Coronado et al., 2014). Davis et al. (Davis et al., 2017) compared screening uptake between groups provided a FIT kit (low health literacy photonovella booklet and video versus standard brochure). In contrast to the findings above, no significant difference between groups were identified. Although Davis et al. (Davis et al., 2017) reported they could not fully identify which intervention component were most effective and participants were not limited to those not up-to-date with screening (Davis et al., 2017). Therefore, for nationwide screening kit information to be understandable for the whole population, information should target low health literacy, including plain language, more pictures and scan codes to animations/narratives explaining information in different ways.

### 4.3 Health professional education and counselling

The HPE studies mixed results showed improvements in awareness may not lead to increased screening. Alternatively, providing screening directly to participants may contribute to higher screening (Boutsicaris et al., 2021, Ou et al., 2019, Ruggli et al., 2019, Christy et al., 2016, McBride and Gesink, 2018, O’Keefe et al., 2018). As five of six studies with moderate-high post-intervention screening rates, provided a screening option to participants. A pharmacist intervention provided FITs directly to 21,596 participants with successful test completions, suggesting this approach is effective to reach more individuals to screen (Ruggli et al., 2019). The study reported almost half of participants would not have screened without the intervention (Ruggli et al., 2019). This is consistent with other education intervention types in this review. Boutsicaris et al. (Boutsicaris et al., 2021) suggested providing FITs may have improved screening following inflatable colon tours. As both Boutsicaris et al. (Boutsicaris et al., 2021) and Portilla-Skerrett et al. (Portilla-Skerrett et al., 2019) had no change in intentions post-tour. Ou et al. (Ou et al., 2019) reported HPE only increased screening due to providing FITs to participants. McBride and Gesink (McBride and Gesink, 2018) and O’Keefe et al. (O’Keefe et al., 2018) suggested providing kits in these Canadian and USA studies reduced barriers to screen, easier to access (transport, costs), convenient and acted as a reminder. These findings are consistent with two reviews (Schliemann et al., 2021, Leach et al., 2021). Schliemann et al. (Schliemann et al., 2021) found more screening uptake with education and providing kits compared to education alone. Leach et al. (Leach et al., 2021) observed a larger effect size in increasing screening among studies which provided free or low-cost screening options. Therefore, providing screening directly to participants can be more effective than education alone.

### 4.4 Lay community health educators’ education or counselling

Majority of LCHEE studies showed statistically significant improvements in awareness. LCHEE with high screening improvements were enhanced education sessions within Filipino American community organisations with organisation allocated educators (Maxwell et al., 2016) and barber LCHEE within a barbershop (Sizer and Conyers, 2022). A nurse educated the barber to provide education to eligible clients during appointments. Contributors to success of these interventions may be removing barriers to access screening and utilising highly trusted community members as educators. This may be due to social connections and support between participants and educators. As many educators were chosen from within the target population/communities. An intervention where children taught families both significantly improved awareness and screening intentions (Parker et al., 2021). This child educator approach is novel in bowel cancer education and could be beneficial for future interventions. A similar approach was utilised for reducing tobacco smoking (Chung et al., 2019). The study educated teenagers about risks and encouraged cessation and found teenagers effectively promoted the information to peers, friends and family (Chung et al., 2019). These interventions may be effective by providing health promotion in often difficult to reach groups (Chung et al., 2019). In contrast, a LCHEE and patient navigation study found no difference in screening but statistically significant changes in knowledge among those who did and did not attend education (Mojica et al., 2016). Mojica et al. (Mojica et al., 2016) found some participants wanted to attend education but never intended to screen due to cost and time. This is consistent Cancer Council Victoria (Cancer Council Victoria, 2021), which suggest some individuals who do not screen can be classified as ‘refusers’, who are aware of screening although do not wish to participate. Indicating an increase in awareness may not lead to an increase in screening among refusers.

### 4.5 Social support

Social support is used within several interventions and may have assisted in screening improvements. Dominic et al. (Dominic et al., 2020) combined education sessions with loved one’s supporting participants to screen. Other interventions had community members or peers involved in group discussions (McBride and Gesink, 2018) or cast members of a theatre play (Friedman et al., 2019), all with promising results. Similarly, the 2016 review (Martini et al., 2016) found education and peer support increased screening motivation although, did not impact screening uptake at 6-months. These approaches may assist higher reach to raise awareness, promote health, and encourage screening by inspiring discussions among family and friends. As James et al. (James et al., 2022) reported individuals mostly prefer to discuss health with individuals they trust. Education interventions could benefit from incorporating social supports or LCHEE from within the target populations to promote success of the intervention.

### 4.6 In-person and virtual education components

Interventions that worked well to improve awareness had in-person components compared to virtual only. A phone versus web-based counselling study found phone counselling increased knowledge and screening more. This in-person approach, along with group discussions or individual education may be due to more opportunities to ask questions (Christy et al., 2020). Virtual interventions can limit this. The Facebook group intervention did not provide such opportunities and found improvements in awareness although no screening change (Key et al., 2020). The intervention may promote health messages to difficult to reach populations although, not encourage screening. Consistently, Yaacob et al. (Yaacob et al., 2020) used a mobile app to improve knowledge and attitudes towards screening. The app was successful to improve knowledge although, not screening attitudes. This may be due to difficulties in changing individuals’ attitudes and behaviours; one needs to be ready for change and their values and beliefs need to align with the behaviour (Yaacob et al., 2020). The Women’s health day, another in-person approach found combining screening and education with other health screening and priorities created a holistic approach and increased screening motivation (McBride and Gesink, 2018). The authors of this review were unable to identify other studies which combined health checks although, future interventions could consider such approach to encourage participation. Art-based education was another useful in-person approach. Friedman et al. (Friedman et al., 2019) reported the theatre play successfully distributed information, with moderate improvements in screening intentions. Similarly, Lofti-Jam et al. (Lofti-Jam et al., 2019) implemented a comedy show about screening for indigenous Australians with good results. Following the show, 88 % of attendees intended to screen and 76 % reported good screening awareness (46 % increase from pre-show) (Lofti-Jam et al., 2019). Art-based education could be a useful education strategy and should be further explored. Interventions attempting to improve awareness could do well to incorporate in-person approaches.

## 5 Strengths and limitations

A strength was the review provided contemporary information regarding awareness and screening education interventions. An eligibility criterion guided the inclusion process, and two authors screened all studies to remove risk of bias. A limitation was the search strategy included only English language studies which may limit findings for some OECD countries. Secondly, although grey literature was eligible in the inclusions, only published articles were identified. Due to human error, misclassification of studies may have occurred during data synthesis. Lastly, due to varied outcome measures between studies, an overall effect size for awareness and screening could not be produced.

## 6 Conclusion

Education types identified to improve bowel cancer awareness and screening were LCHEE, education materials, HPE, mass media and other. LCHEE were most common and effectively improved both awareness and screening. Brochures improved screening, but brochures plus LCHEE were more effective for awareness. A state-wide campaign run multiple times a year may be an effective mass media intervention for screening uptake. Providing screening opportunities with education would encourage screening, by reducing barriers and increasing convenience. Findings within this review could assist education intervention development for bowel cancer awareness and screening.

**Author contributions**.

NG drafted the scoping review protocol, search strategy, data analysis, and manuscript. KO, SL, and MS reviewed the scoping review protocol, search strategy and draft manuscript. All authors were involved in the screening and data extraction.

## CRediT authorship contribution statement

**Nicola Gadd:** Writing – review & editing, Writing – original draft, Software, Methodology, Formal analysis, Conceptualization. **Simone Lee:** Writing – original draft, Supervision, Software, Methodology, Formal analysis, Conceptualization. **Matthew J Sharman:** Writing – review & editing, Writing – original draft, Supervision, Software, Methodology, Formal analysis, Conceptualization. **Kehinde Obamiro:** Writing – review & editing, Writing – original draft, Supervision, Software, Methodology, Formal analysis, Conceptualization.

## Declaration of competing interest

The authors declare that they have no known competing financial interests or personal relationships that could have appeared to influence the work reported in this paper.

## Data Availability

No data was used for the research described in the article.
