# Supplementary material for: Educational interventions to improve bowel cancer awareness and screening in Organisation for Economic Co-operation and Development countries: A scoping review
Source: Prev Med Rep. 2024 Feb 13;39:102653. doi: 10.1016/j.pmedr.2024.102653 (PMC10879007; doi:10.1016/j.pmedr.2024.102653)
Supplement: Supplementary data 1 [file mmc1.docx]

**Educational Interventions to Improve Bowel Cancer Awareness and Screening in Organisation for Economic Co-operation and Development Countries: A Scoping Review.**

APPENDICES

**Appendix A:** Systematic search strategy of four databases for the scoping review.

| ***MEDLINE (via PubMed) searched on 25/03/2022 using keywords and MeSH Terms.***  ***Result = 1,781 records*** |
| --- |
| ("colorectal neoplasms"[MeSH Terms] OR "colorectal cancer"[Title/Abstract] OR "rectal cancer"[Title/Abstract] OR "colorectal adenocarcinoma"[Title/Abstract] OR "bowel cancer"[Title/Abstract]) AND ("early detection of cancer"[MeSH Terms] OR "awareness"[MeSH Terms] OR "faecal occult blood test"[Title/Abstract] OR "faecal immunochemical test"[Title/Abstract] OR "colonoscopy"[Title/Abstract] OR "sigmoidoscopy"[Title/Abstract] OR "cancer screening"[Title/Abstract] OR "FOBT"[Title/Abstract]) AND ("educational status"[MeSH Terms] OR "education"[MeSH Terms] OR "health promotion"[MeSH Terms] OR "promotion"[Title/Abstract] OR "campaign"[Title/Abstract] OR "education intervention"[Title/Abstract] OR "mass media"[Title/Abstract] OR "promotion activity"[Title/Abstract]) |
| ***EMBASE searched on 25/03/2022 using keywords and MeSH Terms. Result = 1,151 records*** |
| exp colorectal cancer/ OR (rectal cancer or bowel cancer or colorectal adenocarcinoma).ab,kf,ti. AND cancer screening/ OR (FOBT or faecal occult blood test or faecal immunochemical test or early detection of cancer or colonoscopy or sigmoidoscopy).ab,kf,ti. OR awareness/ AND education/ OR (promotion or campaign or education activity or education intervention or mass media or promotion activity).ab,kf,ti. |
| ***Web of Science searched on 25/03/2022 using keywords and MeSH Terms. Result = 2,343 records*** |
| (((TS=(colorectal cancer)) OR TS=(bowel cancer)) OR TS=(rectal cancer)) OR TS=(colorectal adenocarcinoma) AND ((((((TS=(cancer screening)) OR TS=(FOBT)) OR TS=(faecal occult blood test)) OR TS=(faecal immunochemical test)) OR TS=(early detection of cancer )) OR TS=(colonoscopy)) OR TS=(sigmoidoscopy) OR TS=(awareness) AND ((((((TS=(education)) OR TS=(promotion)) OR TS=(campaign )) OR TS=(education intervention)) OR TS=(mass media)) OR TS=(promotion activity)) OR TS=(education activity) |
| ***CINAHL searched on 25/03/2022 using keywords and MeSH Terms. Result = 417 records*** |
| (MH colorectal neoplasms OR TI colorectal cancer OR rectal cancer OR colorectal adenocarcinoma OR bowel cancer OR AB colorectal cancer OR rectal cancer OR colorectal adenocarcinoma OR bowel cancer) AND (MH "Cancer Screening") OR TI ( awareness OR FOBT OR faecal occult blood test OR faecal immunochemical test OR early detection of cancer OR colonoscopy OR sigmoidoscopy ) OR AB ( awareness OR FOBT OR faecal occult blood test OR faecal immunochemical test OR early detection of cancer OR colonoscopy OR sigmoidoscopy ) AND MH ( Education OR "Health Education" ) OR TI ( promotion OR campaign OR education activity OR education intervention OR mass media OR promotion activity ) OR AB ( promotion OR campaign OR education activity OR education intervention OR mass media OR promotion activity ) |
| ***Total records: 5,692*** |

**Appendix B:** Data chart used for data extraction of included studies in the scoping review.

| **Scoping Review Details** | |
| --- | --- |
| Scoping review title | Educational Interventions to Improve Bowel Cancer Awareness and Screening in Organisation for Economic Co-operation and Development Countries: A Scoping Review. |
| Scoping review objective | The objective of this scoping review was to identify the design, implementation, and evaluation (where reported) of educational interventions that have been used to improve bowel cancer awareness and screening in Organisation for Economic Co-operation and Development (OECD) countries. |
| Scoping review questions | 1. How have educational interventions to improve bowel cancer awareness and screening been designed and implemented among adults in OECD countries and what were the outcomes?  2. Were there differences in outcomes based on rurality, socio-economic status, cultural diversity, gender, and/or age? |
| **Inclusion/ Exclusion Criteria** | |
| Population | Adults aged above 18 years were included.  Health professionals or high-risk populations of bowel cancer for example, individuals with family history of bowel cancer were excluded. |
| Concept | Studies and reports which included bowel cancer awareness and screening education interventions were included.  Education on bowel preparation for a colonoscopy or studies which did not implement the intervention for example, focus groups or interviews were excluded. Patient reminders were not considered as education. |
| Context | Studies and reports developed in OECD countries in a community setting were included (excluded clinical settings). |
| Types of evidence source | All study designs with reported intervention results and grey literature (government and non-government reports) were included. Review papers and conference abstracts were excluded. |
| **Evidence Source Details & Characteristics** | |
| Author, reference |  |
| Year of publication |  |
| Title |  |
| -Country of origin (where the source was published or conducted)  -Geographical location, if available (where the source was conducted, rural/remote or metropolitan) |  |
| Aim |  |
| Population and sample size (if applicable)  -Participants (number, % female, mean age, ethnicity) |  |
| Methodology (study design) |  |
| Intervention type, comparator, and details of these (e.g., duration of the intervention, intervention category) (if applicable). |  |
| Awareness outcome(s) and details of these (e.g., how measured, was a validated tool used, if available) |  |
| Screening outcome(s) and details of these (e.g., how measured) |  |
| **Details/ Results Extracted from Evidence Source** | |
| Key findings on awareness outcome that relate to the scoping review questions. |  |
| Key findings on screening outcome that relate to the scoping review questions. |  |

# Table adapted from Peters et al. (1).

**Appendix C:** Summary of the characteristics and findings of bowel cancer awareness and screening findings of the included studies (n=62) in the scoping review.

| **Study design & setting** | **Participants & comments** | **Bowel cancer educational intervention** | **Study outcome measures & evaluation methods** | **Bowel cancer awareness findings** | **Bowel cancer screening findings** |
| --- | --- | --- | --- | --- | --- |
| **MASS MEDIA (n=5 studies)** | | | | | |
| ***Public awareness campaign*** | | | | | |
| Torrance et al. (66), 2021, Cohort study,  UK |  | Public awareness campaign via television, bus stop posters and billboards.  2 groups: campaign region and control region. | Knowledge | Knowledge improved significantly for abdominal symptoms bloating (Pre: n=269, 67%; Post: n=297, 74%; p=0.03) and nausea (Pre: n=277, 69%; Post: n=305, 76%; p=0.03) compared to the control group. But not for diarrhoea, discomfort, and other symptoms. |  |
| Katz et al. (82) 2017,  RCT, USA | n=1185; Female: 58%; Caucasian (98.5%); mean age: 62yrs.  Social cognitive theory used to develop study. | Mass media campaign via billboards, posters, and local newspaper articles.  Control: fruit and vegetable campaign.  2 groups: screening campaign and control campaign. | Screening intent.  Colonoscopy, FOBT, sigmoidoscopy. | Above 85% of total participants who saw the billboards reported they were clear, important, and easy to understand. Above 90% of total participants who saw the posters reported they were clear, important, and easy to understand. The newspaper articles were not seen by many participants in either group during the campaign. | The bowel cancer screening billboards led to 2x higher intentions for participants to speak with their doctors about screening compared to the control group. The screening poster participants were more than twice as likely to intend to speak with their doctor about screening compared to the control. The odds of participants completing screening after the interventions (billboards and posters) was not significantly different between the groups (OR from multiple imputations = 0.87, 95%CI: 0.51, 1.50). |
| Durkin et al. (68), 2020, Cross sectional study, Australia |  | Mass media campaign for 7 weeks via television, radio, webpage, and social media advertisements.  2 groups: campaign state and control state | Screening uptake.  FOBT. |  | Screening rates increased during the campaign weeks for the campaign state with a difference of 10.9% compared to the non-campaign weeks (comparison state difference was 2%). There was a significantly higher rate of screening among those who had not screened before compared to previous screeners. The screening increase rose within two weeks after initiating the campaign, peaked during weeks 4-7 and remained high for 4-weeks after the campaign ended. |
| Durkin et al. (87) 2019, Cross sectional study, Australia |  | Mass media campaign for 8 weeks via television, digital and online advertising.  2 groups: campaign and advertising state and campaign only state. | Screening uptake.  FOBT. |  | Both states had an increase in FOBT completed during the campaign (campaign state: adjusted rate ratio 20%, 95%CI: 1.06, 1.35, p<0.01; non-campaign state: adjusted rate ratio 11%, 95%CI: 0.99, 1.24, p=0.087). But the campaign and advertising state had a higher increase in FOBT screening completed during and 2-months post-campaign. |
| ***Facebook campaign*** | | | | | |
| Koivogui et al. (88), 2020, Non-randomised experimental study, France | n=39,900  Targeted to 50-74yrs Facebook users. | Facebook awareness message campaign via promotional messages and videos. | Screening uptake.  FIT. |  | 53.7% (n=298) of participants requested a screening kit, 160 were eligible and 67 (42%) completed the test. 8.9% of the target population clicked on a campaign message or video. |
| **EDUCATION MATERIALS (n=5 studies)** | | | | | |
| ***Brochures*** | | | | | |
| Jo et al. (62)  2017, RCT, USA | n=348; Female: 84%; Korean American (100%); mean age: 61yrs.  Social Cognitive Theory and Transtheoretical model used to develop study. | Education sessions run by LCHEs via flipchart, training manual and brochure about screening.  Control: brochure and 2 education sessions on nutrition and physical activity.  2 groups: LCHE + brochure and brochure only (control) | Knowledge, screening uptake.  Colonoscopy, FOBT, sigmoidoscopy. | Knowledge increased more for the LCHE + brochure group compared to brochure only control at 6-month follow-up (p<0.001). | The two groups had similar significant changes in completing screening at follow-up except for the FOBT where the LCHE + brochure group were more likely to be up to date compared to brochure only. LCHE + brochure group ever under-went FOBT: (23.9% to 35.3%; p=.0005). |
| Nguyen et al. (63) 2017, RCT, USA | n=725; Female: 81%; Chinese (100%); mean age: 62.2yrs. | LCHE run 2 group education sessions and 2 telephone calls.  2 groups: LCHE + brochure and brochure only. | Knowledge, screening uptake.  Colonoscopy, FOBT, sigmoidoscopy. | A significant knowledge increase was reported across 9 measures for LCHEs + brochures and in 6 for brochures only (p<0.002). Knowledge increases were higher across all 9 measures for LCHEs + brochures compared to brochures only. | Both the LCHE + brochure and brochure only groups had an increase in ever been screened although, only LCHEs + brochures had an increase in screening intentions among those who were not up to date with screening. |
| Cuaresma et al. (64) 2018, RCT, USA | n=304; Female: 77%; Pilipino (91%). | Education sessions using brochures and flipcharts, run by LCHEs 2 months apart.  Control group: Education and brochure on nutrition and physical activity for cardiovascular health.  2 groups: Education and control. | Awareness, screening uptake.  Colonoscopy, FOBT, sigmoidoscopy. | Both intervention and control groups had an increase in screening awareness.  Intervention group changes: heard of colon cancer: +5%, p=0.22; heard of FOBT: +8%, p<0.0001; heard of sigmoidoscopy: +39%, p<0.0001; heard of colonoscopy: +9%,  p=0.001.  Control group changes: heard of colon cancer: +1%,  p=0.80; heard of FOBT: +11%, p=0.014; heard of sigmoidoscopy: +10%, p=0.33; heard of colonoscopy: -3%, p=0.41.  Although, slightly higher (not statically significant) increases were observed for the intervention group (Differences between groups, heard of colon cancer: p=0.49; heard of FOBT: p=0.024; heard of sigmoidoscopy: p=0.0014; heard of colonoscopy: p=0.0086). | Screening increased by 9% for the intervention group and 1% for the control group. This difference was not statistically significant. |
| ***National screening kit and brochure*** | | | | | |
| Fransen et al. (79) 2017, Mixed method study, Netherlands | n=127; Female: 68%.  Low health literacy.  Interview about the accessibility of the screening kit and comprehensibility of the text materials. | National FOBT screening package (instructions, brochure, test kit) and invitation letter. | Knowledge.  Colonoscopy, FOBT. | Knowledge about screening improved for 10 out of 16 items after reading the screening kit instructions and brochure compared to baseline. Knowledge remained low for some items measured; bowel cancer risk, test sensitivity and that screening is voluntary. |  |
| ***Photonovella or brochure*** | | | | | |
| Christy et al. (65) 2016, Non-randomised experimental study, USA | n=330; Female: 48%; African American (93%), mean age: 56yrs. | Culturally targeted photonovella booklet or standard brochure and FIT provided.  2 groups: photonovella booklet and brochure. | Awareness, screening uptake.  FIT. | Higher mean (SD) awareness was reported among the brochure group (7.1 (2.0)) compared to the photonovella group (6.8 (2.4)), p=0.149. A significant association was identified between lower awareness and not completing screening. | 86.7% of participants completed the FIT provided. The photonovella group increased screening by 60% and brochure by 56.3% at follow up although, the interventions were not significant determinants for screening. |
| **HEALTH PROFESSIONAL EDUCATION/ COUNSELLING (n=10 studies)** | | | | | |
| ***Physician led presentation*** | | | | | |
| Mukherjea et al. (80), 2020, Non-randomised experimental study, USA | n=103; female: 43%; Hinduism (46%), Islam (24%), Sikhism (23%); mean age: 69yrs. | South-Asian physician led presentation and culturally tailored brochure. | Screening uptake; screening intent.  FOBT. |  | Screening intentions increased following the presentation by 39% among participants who had not screening previously. Among those with previous screening, 84% reported they intent to screen again. |
| Hoffman et al. (81) 2016, Non-randomised experimental study, USA | n=256; Female: 80%; African American (93.1%); mean age: 61yrs.  The CLEAN framework (Culture, Literacy, Education, Assessment, and Networking) used to develop study. | Health professional (surgeons, medical students) networking, presentation, discussion, and question time. | Knowledge.  Colonoscopy. | 75% (n=142) participants agreed that early detection could lead to a cure and 48.2% (n=91) were aware of bowel cancer causes although, 60% (n=113) and 46% (n=87) misunderstood malignancy and metastatic, respectively. |  |
| ***Physician and lay educator led presentation*** | | | | | |
| Cassel et al. (58) 2020, Cross sectional study, USA | n=528; Female: 100%; Hawaiian (100%). | 2 education sessions run by LCHEs and a physician using flipcharts and a slideshow (culturally appropriate information). | Knowledge, screening intent.  FIT. | 92% (n=137) of participants over 50yrs learnt something about bowel health. | 76% agreed to do FIT and 79% were up to date with screening following the education sessions. The group education was shown to help encourage other participants to complete screening. The study reported that handling stools is considered forbidden in Hawaiian culture. |
| ***Physician and nurse led education*** | | | | | |
| Chow et al. (57) 2020,  Cohort study, Canada; rural/remote | n=333; First nations people (100%).  Flipchart used culturally appropriate information. | Physician and nurse education during stays at lodge for rural patients awaiting health visits using an education toolkit (flipbook). Initially used model of the colon. | Screening intent.  FOBT. | 99% of participants reported that the health professionals explained the information in a simple way to understand. 98% (n=386) felt they were provided enough information about screening. | 32% (n=106) of eligible participants were provided the FOBT. |
| Naguib et al. (61) 2017, Case report, UK | n=772; Female: 54%; median age: 55yrs. | Mobile bus clinic run by health providers via sigmoidoscopy and brochures. | Screening uptake.  Colonoscopy, FIT, sigmoidoscopy, barium enema, CT colonoscopy. | Bowel bus clinic led to health professionals discussing bowel cancer signs and symptoms with 772 community members. | 32% (n=244) of participants were screened on the bus. The bus led to a reduction in the wait times by 4.6weeks for routine referrals to the hospital colorectal unit after six months. 92% reported they would use the bowel bus clinic services again and 96% would recommend it to a friend. |
| ***Pharmacist led counselling*** | | | | | |
| Holle et al. (56), 2020, Cross sectional study, USA | n=16; female: 56.2%; African American (62.5%). | Pharmacist’s counselling and provided FIT with verbal and written instructions. | Knowledge, screening uptake.  Colonoscopy, FIT. | Knowledge scores increased significantly between baseline and follow-up in both groups (mean difference: Intervention group: 0.48, 95%CI: 0.21, 0.75. Control group: 0.48, 95%CI: 0.21, 0.74). There was no difference between the groups (mean difference: 0.00, 95%CI: -0.38, 0.38). | 88% (n=7) of eligible participants completed the FIT following the counselling. |
| Ruggli et al. (59) 2019, Non-randomised experimental study, Switzerland | n=23,024 | Pharmacist led campaign (6 weeks) via education and with FIT provided. Positive FIT results or those with risk factors were referred to a physician. | Screening uptake.  Colonoscopy, FIT. |  | 97% (n=21,596) of participants completed the FIT and 3% were referred to physicians. 47% (n=10,464) of participants reported they would not have completed the screening without the campaign. All participants with positive results were referred to a physician. |
| ***Nurse practitioner and clinic staff*** | | | | | |
| O’Keefe et al. (86) 2018, Non-randomised experimental study, USA | University employees | A workplace health communication event and an employee wellness program via an information postcard, merchandise and provided FIT or scheduled colonoscopy.  2 groups: University of Alabama and University of South Alabama. | Screening uptake.  FIT. |  | There was a 35% increase in screening among participants following the employee wellness program/event with >70% return rate of FIT at both university sites (site 1: 72%, n=62; site 2: 76%, n=111). Employees reported that the on-site FIT clinic to pick-up and drop-off the tests was very convenient and encouraged screening. |
| ***Community health workers*** | | | | | |
| Briant et al. (55) 2018, Non-randomised experimental study, USA; rural/remote | n=101; Female: 70%; Hispanic 99% | Bilingual health workers run 2 home health parties using flipcharts. Eligible participants provided FOBT. | Awareness, screening intent.  Colonoscopy, FOBT, sigmoidoscopy. | Participants increased their awareness about screening following the home health parties (change score: 0.63; p<0.0001). | Screening increased significantly following the parties with 86.7% participants completing FOBT and only 3 participants were non-adhered at follow-up. |
| ***Nurse and psychologist counselling*** | | | | | |
| Denis et al. (60) 2017, RCT, France | n=48350; Female: 50% | Telephone counselling for screening run by health providers (nurses and a psychologist) via motivational interviewing or a computer system prompted questions for counsellor + recall letter.  Control: usual care; mail FOBT + untailored recall letter.  3 groups: computer assisted telephone counselling, telephone counselling only, control. | Screening uptake.  FOBT. |  | Following the counselling interventions, screening intentions (accepted to screen) had increased from 18.5% to 78.2% and those who were undecided had reduced from 46.3% to 10.8%. There was minimal change in those who decided to refuse the screening (10% to 10.8%). No difference was observed between the two intervention groups with screening intentions after the counselling. The main determinant for screening participation was screening history, with participants more likely to screen if they had screened before (OR:11.2) or were first time screening (OR: 2.2) compared to those who had never screened. |
| **LAY COMMUNITY HEALTH WORKER EDUCATION/ COUNSELLING (n=28 studies)** | | | | | |
| Jenkins et al. (40), 2022,  Non-randomised experimental study, USA | n=515; female: 73.4%; Caucasian (96%) | Faith-based education sessions x3 and motivational interviewing x2 by local LCHEs.  2 groups: early and delayed interventions (3m post). | Knowledge.  Colonoscopy, FOBT. | Knowledge improved moderately from baseline score (+0.17, p= 0.06). Significant correlation with a reduction in psychological barriers. Faith-based LCHEs were shown to be effective for cultural preferences. |  |
| Gray et al. (44), 2021,  Cross sectional study, UK | n=137; female: 50.4%.  Individuals with learning disabilities. | Peer-led education session. | Knowledge, screening intent.  FIT. | Knowledge improved following the session.  Do you know what bowel screening is? YES: Pre: n=27, 19.7%; Post: n=137, 100%.  Do you know how to do the test? YES: Pre: n=21, 15.3%; Post: n=130, 95%.  Can you help your bowel to be healthy by being active? YES. Pre: n=43, 31.4%. Post: n=128, 93.4%.  Can you help your bowel to be healthy by eating healthy food? YES. Pre: n=75, 54.7%. Post: n=128, 93.4%. | Screening intentions improved following the session (Pre: n=53, 38.7%; Post: n=119, 87%). 98.5% of participants reported they would ask for help to do the test following the session. |
| Dominic et al. (43) 2020, RCT, USA | n=264; Female: 55%; Latino (100%).  Theory of Planned behaviour used to develop study. | Education session, instructions to use FIT kit, brochure, and social support.  2 groups: Social support and no social support. | Knowledge, screening uptake.  Colonoscopy, FIT. | Statistically significant positive changes in 3 out of 12 questions about beliefs (including screening intentions).  A change in bowel habits is a symptom: p=0.0008, n=208.  Screening can identify early-stage BC: p=0.0056, n=211. | Statistically significant positive changes to intend to screen (p=0.0084, n=207). 66% (n=103) of the social support intervention group completed the FIT and 47.2% (n=51) of the control group. The intervention group were 2.17x more likely to screen than the control group. 60% of participants attended the program with loved ones. |
| Christy et al. (52) 2020, RCT, USA | n=891; Female: 100%; Caucasian (88.2%); mean age: 58.6yrs.  Health Belief Model, Transtheoretical Model, and Likelihood Persuasion Behavioural Theory used to develop study. | Web-based interactive computer program and phone counselling guided by a computer program.  4 groups: Web-only, phone only, web + phone and usual care. | Screening uptake.  Colonoscopy, FOBT, FIT. | Phone counselling had better improvements than web program, but phone counselling had more opportunities for participants to ask questions. | Phone counselling group had 52.5% (n=113) participants who completed screening at follow-up compared to web-only (22.7%, n=38) and web + phone (44.4%, n=98) and usual care (24.6%, n=78). |
| Warner et al. (42) 2019, Cross sectional study, USA | n=265; Female: 80%; Latino (100%) | 2 workplace education sessions run by LCHEs via phone, face-to-face and support to schedule screening. | Knowledge, screening uptake.  Colonoscopy, FIT, sigmoidoscopy. | Knowledge increased following the workplace education sessions. Knowledge of the age to begin screenings: 49.8% to 80.7%; p=0.001.  Knowledge of the frequency of bowel cancer screening: 72.1% to 84.5%; p<0.001. | Screening intentions increased following the workplace education sessions. Screening through FIT increased from 13.8% to 56.9% from the intervention although, colonoscopy and sigmoidoscopy were not changed. |
| Ou et al. (45) 2019,  Non-randomised experimental study, USA | n=307; Female: 80%; Hispanic (100%) | Education session run by LCHEs and support to schedule screening (FIT). | Knowledge, screening uptake.  Colonoscopy, FOBT, FIT, sigmoidoscopy. | The study found that awareness did not lead to the screening uptake for Hispanic participants, but the education did support uptake when combined with providing participants with the FIT. | There was a 26% increase in screening uptake following the intervention which may be attributed to the LCHEs providing FIT to participants. |
| Rafie et al. (46), 2020, Non-randomised experimental study, USA | n=188 | Worksite awareness program via information sessions, screening pledge and peer champion activities. | Screening uptake.  Colonoscopy, FOBT, FIT, sigmoidoscopy. |  | Screening uptake increased by 20.6%, with 42% of participants completing the screening and 65% discussing with others about screening. |
| Cuaresma et al. (64) 2018,  RCT, USA | n=304; Female: 77%; Pilipino (91%) | Education sessions using brochures and flipcharts, run by LCHEs 2 months apart.  Control group: Education and brochure on nutrition and physical activity for cardiovascular health.  2 groups: Education and control. | Awareness, screening uptake.  Colonoscopy, FOBT, sigmoidoscopy. | Both intervention and control groups had an increase in screening awareness.  Intervention group changes: heard of colon cancer: +5%, p=0.22; heard of FOBT: +8%, p<0.0001; heard of sigmoidoscopy: +39%, p<0.0001; heard of colonoscopy: +9%,  p=0.001.  Control group changes: heard of colon cancer: +1%,  p=0.80; heard of FOBT: +11%, p=0.014; heard of sigmoidoscopy: +10%, p=0.33; heard of colonoscopy: -3%, p=0.41.  Although, slightly higher (not statically significant) increases were observed for the intervention group (Differences between groups: heard of colon cancer: p=0.49; heard of FOBT: p=0.024; heard of sigmoidoscopy: p=0.0014; heard of colonoscopy: p=0.0086). | Screening increased by 9% for the intervention group and 1% for the control group. This difference was not statistically significant. |
| Woodruff et al. (49) 2017, Cross sectional study, USA | n=185; Female: 70%; non-Hispanic Black (75.7 %); mean age: 61yrs | 23 community outreach education events via group education, brochures, or an inflatable walkthrough colon. | Screening uptake.  Colonoscopy, FOBT, FIT, sigmoidoscopy. | 74% (n=73) of participants correctly knew their screening status, 12% knew they were due for screening and 61% knew they were not due for screening following the community outreach events. | ~50% of participants who were due for screening completed it by 3-month follow-up. |
| Tong et al. (48) 2017, RCT, USA | n=329; Female: 74%; Hmong American (100%); mean age: 60.4yrs.  Social Cognitive Theory and Transtheoretical model used to develop study. | LCHEs run 2 group education sessions using a flipchart and follow-up telephone calls to answer questions.  Control: LCHE education about cardiovascular health and follow-up telephone calls about diet.  2 groups: education sessions and control. | Knowledge, screening uptake.  Colonoscopy, FOBT, sigmoidoscopy. | Intervention group had a greater change in bowel cancer awareness at follow-up than the control group (heard about FOBT: intervention: 69.6% to 90.7%; control 74.4% to 79.8%, p=0.0017).  The increase of awareness was associated with an increase in the odds of ever screening (OR: 1.29, 95%CI: 1.08, 1.55 increased for every point increase in knowledge). | Intervention group had a greater change in screening at follow-up than the control group (being up to date (p<0.0001). The intervention group had a higher change in ever screening compared to the control (p=0.068). |
| Nguyen et al. (63) 2017, RCT, USA | n=725; Female: 81%; Chinese (100%); mean age: 62.2yrs | LCHE run 2 group education sessions and 2 telephone calls.  2 groups: LCHE + brochure and brochure only. | Knowledge, screening uptake.  Colonoscopy, FOBT, sigmoidoscopy. | A significant knowledge increase was reported across 9 measures for LCHEs + brochures and in 6 for brochures only (p<0.002). Knowledge increases were higher across all 9 measures for LCHEs + brochures compared to brochures only. | Both the LCHE + brochure and brochure only groups had an increase in ever been screened although, only LCHEs + brochures had an increase in screening intentions among those who were not update with screening. |
| Molokwu et al. (41) 2017, RCT, USA | n=784; Female: 78%; Hispanic (98%).  Social cognitive theory and health belief model used to develop study. | Culturally and low-literacy tailored education session run by LCHEs via video and flipchart.  4 groups: education session only, video only, education session + video, control. | Knowledge.  Colonoscopy, FOBT. | The three intervention groups were analysed together due to previous study finding no difference in the education delivery methods (video only, LCHE only or LCHE + video). The intervention group significantly increased knowledge at 6-month follow-up compared to the control group (0.74 vs 0.18, p<.0001). |  |
| Mojica et al. (53) 2016, Cross sectional study, USA | n=535; Female: 100%; Latino (100%); mean age: 46yrs | Bilingual LCHE education sessions and patient navigation for screening via bilingual flipcharts (Spanish, English), posters and models. | Knowledge, screening uptake.  FOBT. | Knowledge significantly changed following the LCHE education about screening and early detection of BC. Participant’s knowledge of stool tests starting >50yrs improved from 19% to 70% and the frequency of stool tests were correctly answered by 42% to 75% of participants after the education. | Eligible participants were not more likely to screen following the education session than those who did not attend the session (6.7% vs. 12.1%, p=0.155, after adjustment OR: 0.64, 95%CI: 0.24, 1.71). |
| Maxwell et al. (50) 2016, RCT, USA | n=673; Female: 62%; Filipino American (100%); mean age: 61yrs.  59% of the organisations who participated in the study had previously been exposed to bowel cancer studies. | LCHE group education session and provided FOBT. Enhanced education provided 3 extra sessions, had more LCHEs, 6 additional promotion activities and a leader report additional findings back to group.  2 groups: basic education and enhanced education. | Screening uptake.  FOBT. |  | Screening increased following the basic and enhanced education interventions. There was an 83% difference between groups screening uptake with more enhanced group participants completing screening at 6-month follow up. |
| Jo et al. (62)  2017, RCT, USA | n=348; Female: 84%; Korean American (100%); mean age: 61yrs.  Social Cognitive Theory and Transtheoretical model used to develop study. | Education sessions run by LCHEs via flipchart, training manual and brochure about screening.  Control: brochure and 2 education sessions on nutrition and physical activity  2 groups: LCHE + brochure and control. | Knowledge, screening uptake.  Colonoscopy, FOBT, sigmoidoscopy. | Knowledge increased more for the LCHE + brochure group compared to brochure only control at 6-month follow-up (p<0.001). | The two groups had similar significant changes in completing screening at follow-up except for the FOBT where the LCHE + brochure group were more likely to be up to date compared to brochure only. LCHE + brochure group ever under-went FOBT: (23.9% to 35.3%; p=.0005). |
| ***Guided tour through inflatable colon*** | | | | | |
| Boutsicaris et al. (33), 2021, Non-randomised experimental study, USA | n=85; female: 69% | Education session with PowerPoint presentation and a 20min guided tour through an inflatable colon.  2 groups. | Knowledge, screening intent.  FIT. | Knowledge increased by 72% for the total group. Education session with PowerPoint (50mins education) had a knowledge increase by 87.5%. The inflatable colon (20mins education) increased knowledge by 65%. The odds of a change in knowledge were nearly 6x more in the PowerPoint group compared to the inflatable colon group although, the inflatable colon was the more attractive method for community members. Change in knowledge did not have a statistically significant association with screening intent. | There was no statistically significant change in screening intentions (Intentions: Pre: n=53, 38.7%; Post: n=119, 87%). This study suggested providing onsite screening kits may improve the screening intent. |
| Miguel et al. (34), 2020, Non-randomised experimental study, USA | n=294; female: 60%; African American (66.7%) | A 7min guided tour through an inflatable colon. | Knowledge, screening intent.  Colonoscopy, FOBT, sigmoidoscopy. | Knowledge increased by 30% following the tour through the inflatable colon, with 99.6% of participants reported as somewhat or very knowledgeable post-tour. 99.6% of participants found the tour an effective way to educate about bowel cancer and participants reported they were more likely to discuss with others about bowel cancer than before the tour. | 79.2% participants were very likely and 18% somewhat likely to intend to screen post-tour; a 12.1% increase in intentions to screen. |
| Portilla-Skerrett et al. (35) 2019, Non-randomised experimental study, USA | n=154; Hispanic (100%) | A guided tour through an inflatable colon in 2 community events. | Awareness, knowledge, screening intent.  Colonoscopy, FOBT, FIT, sigmoidoscopy. | A significant increase in knowledge and awareness (p<0.0001). CRC mainly affects males (p<0.0001), CRC is a tumour that affects the large intestine (p<0.0001), and colonoscopy should be done at age 50 and at age 40 if there is family history of CRC (p=0.003). | There was no change observed in intentions to screen (Likelihood to get screened: Very unlikely/ Somewhat unlikely/Neutral: Pre: n=10, 6%; Post: n=10, 6%; Very likely/Somewhat likely: Pre: n=144, 94%; Post: n=144, 94%  p=0.999). |
| Molina et al. (36) 2018, Cross sectional study, USA; rural/remote | n=1432; Female: 73%; Hispanic (67%) | A guided tour through an inflatable colon at 47 community events (health fairs, tribal gatherings) run by bilingual LCHEs (provided English, Spanish culturally appropriate information).  2 groups: New Mexico State and Washington. | Knowledge, screening intent. | More participants >50yrs reported no or less change in knowledge than an increase in knowledge following the tour. Washington reported an increase in knowledge among 24% (n=225) of participants and New Mexico State reported among 14% (n=68) of participants. Washington had more participants than New Mexico State (n=938 and n=485, respectively). | Participants who were more likely to intend to screen with a change in knowledge was similar between the two groups (Mean, SE: New Mexico: less/equally likely: 2.77, 0.08; more likely: 2.49, 0.09; OR: 0.88; 95%CI: 0.75, 1.03; p=0.11.  Washington: less/equally likely: 2.33, 0.08; more likely: 2.45, 0.10; OR: 1.01; 95%CI: 0.93, 1.10; p=0.76). |
| ***Church setting*** | | | | | |
| Maxwell et al. (39), 2020, Non-randomised experimental study, USA | n=163; female: 62%; African American (100%); mean age: 59yrs | LCHE individual counselling in churches, culturally tailored printed information and reminder calls. | Knowledge, screening uptake.  Colonoscopy, FOBT. | Knowledge about screening was low to moderate at follow-up, with 20% knowing colonoscopy is recommended every 10yrs and 61% knowing stool tests are recommended annually. Knowledge of screening was higher among those who screened following the intervention and not associated with baseline knowledge or the counselling intervention. | 28% (n=46) of participants completed screening following counselling. |
| Maxwell et al. (38) 2019, RCT, USA | n=338; Female: 62%; African American (100%); mean age: 60yrs | Individual education and counselling run by LCHEs using a script. Run in a church. | Screening uptake. Colonoscopy, FOBT, sigmoidoscopy. | 91% (n=168) of participants who discussed screening with the LCHEs stated they had learnt something important and 98% felt comfortable asking questions. 94% would refer the LCHE to a friend and 83% would attend a session on another topic. | Participants were more likely to have completed screening at follow-up if they had done a colonoscopy previously (Baseline colonoscopy YES: 45% vs NO: 28%, p=0.006). |
| Elder et al. (47) 2017, RCT, USA | n=436; Latino (100%) | LCHE run 6 education sessions via videos and handouts in a church. LCHE provided 2 motivational interview telephone calls and accompanied to screening appointments where necessary.  Control: Physical activity education.  2 groups: LCHE screening education and control. | Knowledge, screening uptake.  Colonoscopy, FOBT, sigmoidoscopy. |  | Screening uptake increased for both groups but did not significantly increase for the LCHE screening education compared to the LCHE physical activity education control.  FOBT rates: Intervention group: Pre: 15%, Post: 25%.  Control group: Pre: 13%, Post: 20%.  Colonoscopy/sigmoidoscopy screening rates: Intervention group: Pre: 37%, Post: 53%.  Control group: Pre: 31%, Post: 40%. |
| Leone et al. (51) 2016, RCT, USA | n=955; Female: 69%; African American (100%); mean age: 63yrs.  Social cognitive theory, social support models and the health belief model used to develop study. | Church wellness program with events, LCHE peer counselling via telephone call motivational interviewing and mailed tailored newsletters about screening and physical activity.  Control: Fruit and vegetable promotion program.  2 groups: Wellness program and control. | Screening uptake.  Colonoscopy, FOBT, sigmoidoscopy, double contrast barium enema, virtual colonoscopy. |  | The were no statistically significant differences in screening uptake for the two groups with a 33% and 33.7% increase in screening for those not up to date was reported for the bowel cancer program and control program, respectively. LCHE counselling did not significantly impact screening for participants with 52.2% of participants who screened received counselling and 32.8% did not receive counselling. |
| Holt et al. (37) 2019, RCT, USA | n=375; Female: 68%; African American (100%); mean age: 55yrs | 3 education workshops (at 3, 12, 24months) run by LCHEs in churches.  2 groups: In-person workshop and online workshop. | Knowledge, screening uptake.  Colonoscopy, FOBT, sigmoidoscopy. | Workshop 3 (at 24months) was the only workshop to have a significant group difference in knowledge with the online workshop group reporting higher knowledge than the in-person workshop group. Although, the online workshop group had a higher baseline knowledge compared to the in-person workshop group. 92% participants reported speaking to their friends/family about the program by workshop 2. | Participants who attended all three workshops were more likely to have completed the colonoscopy (OR: 2.14) or FOBT (OR: 2.67) compared to those who only attended one workshop. |
| ***Lay educator and physician education*** | | | | | |
| Cassel et al. (58) 2020, Cross sectional study, USA | n=528; Female: 100%; Hawaiian (100%) | 2 education sessions run by LCHEs and a physician using flipcharts and a slideshow (culturally appropriate information). | Knowledge, screening intent.  FIT. | 92% of participants over 50yrs learnt something about bowel health. | 76% agreed to do FIT and 79% were up to date with screening following the education sessions. The group education was shown to help encourage other participants to complete screening. The study reported that handling stools is considered forbidden in Hawaiian culture. |
| ***Children teaching their families*** | | | | | |
| Parker et al. (54), 2021, Non-randomised experimental study, UK | n=20.  School students learnt about bowel cancer and then taught their families and guests. Students were able to interview bowel cancer patients. | Live event run by students with videos and information stands. | Awareness, screening intent.  FOBT. | 95% (n=19) of families reported the event successfully improved their awareness of BC. | 100% (n=20) reported they are more likely to undergo a screening test following the event. |
| ***Barber in barbershop*** | | | | | |
| Sizer and Conyers (84) 2022, Non-randomised experimental study, USA | n=13; Female: 0%; African-American (100%); mean age: 53yrs.  Barbers were trained by nurse practitioners. | Barbers provided education to patrons eligible for screening and willing patrons were referred to book a colonoscopy or FIT. | Screening intent.  Colonoscopy, FIT. |  | 70% (n=9) participants booked a colonoscopy following the barber provided education. |
| ***University survey centre staff*** | | | | | |
| Champion et al. (85) 2018, RCT, USA | n=1196; Female: 100%; Caucasian (86.3%), African American (10.4%); mean age: 59yrs | Web-based program messages or telephone counselling.  Control: usual care.  4 groups: Web-only, phone-only, web + phone and control. | Screening uptake. |  | The phone only and phone + web-based program groups had significantly higher screening uptakes at 6-month follow up compared to usual care (p=0.0001). The three intervention groups were significantly better at increasing screening intentions compared to usual care (Web: p=0.0140, phone: p=0.0057, Web + phone:  p=0.0032). |
| **OTHER (n=19 studies)** | | | | | |
| ***Video*** | | | | | |
| Lucas et al. (73), 2021, RCT, USA | n=457; female: 74.4%; African American (100%).  Theory of Planned Behaviour framework used to measure outcomes. | Online video module with message manipulation, provided FIT.  2 groups: Gain framed video messages and loss-framed video messages. The 2 groups were randomly allocated to the standard or culturally tailored messages. | Screening intent and uptake.  Colonoscopy, FIT, sigmoidoscopy. |  | Loss-framed messages were more favourable at improving screening intent when the message was culturally tailored compared to the standard loss-framed message.  The loss-framed messages were more effective at improving screening intentions compared to the gain-framed messages. (Mean (SD): Loss: 6.12 (0.838); Gain: 5.96 (0.900), d=0.184).  Culturally tailored messages were more effective than standard messages to improve screening intent. (Mean (SD): Culturally tailored: 6.11 (0.818); Standard: 5.97 (0.923), d=0.161).  292 participants received the FIT kit, and 56 participants completed it. No statistically significant differences were identified between groups in screening uptake. |
| Carcioppolo et al. (67) 2020, RCT, USA | n=186; Female: 67.8%; Caucasian (82.7%); mean age: 55.9yrs | Messaging with fear-based statements about colonoscopy.  2 groups: mixed humour and fear message intervention and fear message control. | Screening intent.  Colonoscopy. |  | Humour and fear intervention was shown as more effective for those who had cancer worries whereas the fear messaging was more effective for those with intentions for colonoscopy. Response efficacy scores:  Fear (mean (SD)): 6.28 (0.87).  Humour + fear (mean (SD)): 5.83 (1.03). |
| Nakajima et al. (70) 2022, Non-randomised experimental study, USA | n=31; Female: 0%; Somali (100%); mean age: 61yrs | Culturally tailored video featuring a Somali-faith leader and Somali doctor and 2 post-video workshop discussion. | Knowledge. | 93.5% (n=29) participants reported they learnt from the video and 3.2% were unsure. 93.5% participants reported they somewhat/very much understand about bowel cancer following the video, compared to 58.1% at baseline. Participants reported the video was helpful to improve their knowledge during the post-video workshop discussion. >90% reported they would recommend screening to others following the video. |  |
| ***Facebook promotion*** | | | | | |
| Lee-Won et al. (72) 2017, RCT, USA | n=140; Female: 50%; Caucasian (90%); mean age: 60yrs | Facebook message promotion via shared blog post with specific message framing.  4 groups: gain-framed + high virality metric, gain-framed + low virality metric, loss-framed + high virality metric and loss-framed + low virality metric. | Screening intent.  Colonoscopy. |  | The targeted messaging loss-framed + high virality metrics had a positive association with colonoscopy intentions through evoking fear. |
| ***Audio recording*** | | | | | |
| Kennedy et al. (89) 2018, RCT, USA | n=442; Female: 100%; African American (100%) | Audio recording listened to in-person with a group in a church.  2 groups: culturally tailored fictional narrative audio (drama/comedy) and expert interview audio. | Screening intent.  Colonoscopy, FOBT, sigmoidoscopy. |  | Participants were more likely to intend to do the FOBT when they heard the narrative compared to the expert interview audio (difference: post-30, F (1, 440) =9.82, p=0.0018).  Screening intentions did not differ between groups for other screening methods. |
| ***Online education module*** | | | | | |
| Lucas et al. (91) 2018, RCT, USA | n=132; Female: 72%; African American (100%).  Theory of Planned behaviour used to develop study. | Online education module with manipulated messaging.  4 groups: loss-framed messaging, loss-framed message + culturally targeted message, gain-framed messaging and gain-framed messaging + culturally target message. | Screening intent. |  | There were no differences in screening intentions for participants with low identity as an African American within the two groups with the online module using loss-framed messaging (messaging: mean (SD) = -0.37 (0.93), messaging + culturally targeted: mean (SD): -0.38 (0.35), d=0.01).  Participants who had a high identity as an African American within these two groups had higher intentions to screen following the online module. The standard gain-framed messaging improved screening intentions more among high identity African Americans compared to the standard loss-framed messaging. Although, high identity participants had most increased screening intentions when observed the culturally targeted loss-framed online module. |
| Lucas et al. (90) 2016, RCT, USA | n=182; Female: 73%; African American (73%).  Theory of Planned Behaviour used to develop study. | Online education module with message manipulation.  Control: Caucasian participants without culturally targeted message.  4 groups: loss-framed messaging, loss-framed message + culturally targeted message, gain-framed messaging and gain-framed messaging + culturally target message. | Screening intent. |  | The screening intentions were higher for Caucasian control group loss-framed messaging compared to the African American group gain-framed messaging.  African American:  intentions: mean: 5.52; SD: 1.27; Item 1: p=0.53; Item 2: p=0.44; Item 3: p=0.23.  Caucasian: Intentions: Mean: 4.88; SD: 1.86; Item 1: p=0.63; Item 2: p=0.65; Item 3: p=0.34.  Gain-framed messaging most impacted African American participants screening intentions although, both loss and gain message framing did not significantly impact the African American participants intentions to screen. The loss-framed messaging had an increase in perceived racism but was lessened by the culturally targeted message. |
| ***Facebook group*** | | | | | |
| Key et al. (83), 2020, Cross sectional study, USA; rural/remote | n=56; female: 66%; Caucasian (100%); mean age: 58yrs | Facebook group for members only with 3 daily posts (polls, videos, scenarios, recipes, bowel cancer information), weekly challenges and prizes. | Screening uptake.  Colonoscopy, FOBT. | Participants reported they learnt how to reduce their risk of bowel cancer following the Facebook group. | No change in screening was reported; 2 participants reported completing screening since the beginning of the study. |
| ***Decision aid*** | | | | | |
| Gabel et al. (76), 2020, RCT, Denmark | n=1679; female: 59%; Danish (97%); mean age: 64yrs | A web-based decision aid was sent a few days after receiving screening reminder.  2 groups: decision aid and control. | Knowledge, screening uptake.  FIT. | Knowledge increased for both groups at follow up  (Mean difference: intervention: 0.48, 95%CI: 0.21; 0.75; control: 0.48, 95%CI: 0.21, 0.74).  Although, no difference was identified between groups (mean difference: 0.00, 95%CI: -0.38, 0.38). | The screening uptake was 7.6% higher in the decision aid group compared to the control group. |
| Woudstra et al. (77) 2019, Cross sectional study, Netherlands | n=81; Female: 55%; mean age: 54yrs | A decision aid about type of screening.  2 groups: low health literacy group and adequate health literacy. | Knowledge, screening intent.  Colonoscopy, FOBT, FIT. | The adequate health literacy participants had improvements in knowledge about screening (Low health literacy (mean (SD)): Pre: 7.72 (2.58); Post: 9.11 (2.21), p=0.013. Adequate health literacy (mean (SD)): Pre: 8.91 (1.93); Post: 9.65 (1.97), p=0.030).  There was no difference between groups for knowledge about bowel cancer after the decision aid (Low health literacy (mean (SD)): Pre: 4.40 (1.26); Post: 4.89 (1.23), p=0.131.  Adequate health literacy (mean (SD)): Pre: 4.70 (1.23); Post: 4.93 (1.25), p=0.195). | There were no differences between groups and the intentions to screening following the decision aid (Low health literacy (mean (SD)): Pre: 4.11 (1.16); Post: 4.34 (1.08), p=0.306.  Adequate health literacy (mean (SD)): Pre: 4.52 (0.72); Post: 4.48 (0.86), p=0.672). |
| Housten et al. (78), 2020, RCT, USA | n=187; female: 63%; African American (70%); median age: 58yrs | Presentation in varied formats.  3 groups: audio booklet with static graphics; video with static graphics; and video with animated graphics. | Knowledge.  Colonoscopy, FOBT, sigmoidoscopy. | There was no advantage to amination compared to static materials in improving knowledge (mean (SD)).  Audio booklet: 66.8 (16.4) vs. static video: 62.0 (20.4), p=0.15.  Audio booklet: 66.8 (16.4) vs. animated video: 65.0 (18.6), p=0.56. | 88.8% (n=166) participants intend to screen in the future. |
| ***A theatre play*** | | | | | |
| Friedman et al. (75) 2019, Cross sectional study, USA | n=110; Female: 72%; African American (100%).  Culturally appropriate and cast members were from the local community. | A theatre play ‘Rise Up, Get Tested, and Live’ with a booth to schedule screening and group discussion with local gastroenterologist about screening. | Knowledge, screening intent.  Colonoscopy, FOBT. | Knowledge increased about bowel cancer and screening among participants following the play.  CRC was preventable: Pre: 78.1% (n=75); Post: 93.6% (n=103)  CRC screening age: Pre: 37.5% (n=36); Post: 64% (n=71), p=0.001.  Colonoscopy is the best test to check for polyps in the colon and rectum: Pre: 75.0% (n=72); Post: 94.6% (n=104), p=0.001. | 60% (n=65) reported they intent to be screened and 86.4% (n=95) reported they will continue to be screened as advised by their health provider following the play. |
| ***Women’s health day*** | | | | | |
| McBride and Gesink (74) 2018, Non-randomised experimental study, Canada | n=75; Female: 100%.  Combined with other health screening in same day. | Women’s health day via bus trip to screening service, health education and shopping. | Knowledge, screening uptake.  FOBT. | 73% (n=55) of participants felt they understood the content covered and 52% reported they knew how to book a screening test. | 52% (n=16) of eligible women completed FOBT screening and 29% (n=9) screened for the first time. Intentions to regularly screen increased by 12%. Combining the screening with other health activities increased the motivation to do the FOBT screening. |
| ***Text message*** | | | | | |
| Alber and Glanz (71) 2018, RCT, USA | n=360; Female: 63%; Caucasian (90.8%); mean age: 61yrs | Cancer screening testimonial text messages about an individual’s screening status.  Control: no text message.  4 groups: 3 text message groups and control. | Screening intent.  Colonoscopy, FOBT, sigmoidoscopy. |  | Above 50% of all participants in all four groups (including the control) were planned/thinking about doing screening following the text message intervention. The text message intervention may not increase the intentions to screen compared to no text message. |
| ***Online message*** | | | | | |
| Neil et al. (92) 2022, RCT, USA | n=738; Female: 56%; Caucasian (77%); mean age: 61yrs | Messaging manipulation on risk factors.  Control: Message on healthy eating and exercise.  3 groups: tailored messaging, targeted messaging, and control. | Screening intent.  Colonoscopy, FOBT, sigmoidoscopy. |  | The tailored online message led to higher screening intentions compared to targeted message and control (83.3%, 79.2% and 70.7% respectively) although, not significant. |
| Champion et al. (85) 2018, RCT, USA | n=1196; Female: 100%; Caucasian (86.3%), African American (10.4%); mean age: 59yrs | Web-based program messages or telephone counselling.  Control: usual care.  4 groups: Web-only, phone-only, web + phone, and control. | Screening uptake. |  | The phone only and phone + web-based program groups had significantly higher screening uptakes at 6-month follow up compared to usual care (p=0.0001). The three intervention groups were significantly better at increasing screening intentions compared to usual care (Web: p=0.0140, phone: p=0.0057, Web + phone:  p=0.0032). |
| ***Patient navigation*** | | | | | |
| Fernandez et al. (32) 2022, RCT, USA | n=1554; Female: 94% African American (43.8%); mean age: 42yrs.  Social cognitive theory used to develop study. | Telephone patient navigation and referral to a referral database for screening.  2 groups: telephone navigation + referral and referral only. | Screening uptake.  Colonoscopy, FOBT, sigmoidoscopy. |  | The patient navigation + referral participants (33%) were more likely to screen than the referral only (23.1%) group although not statically significant (p=0.144). OR = 1.80; 95%CI: 0.90, 3.59; p=0.095. |
| ***Newsletter*** | | | | | |
| Leone et al. (51) 2016, RCT, USA | n=955; Female: 69%; African American (100%); mean age: 63yrs.  Social cognitive theory, social support models and the health belief model used to develop study. | Church wellness program with events, LCHE peer counselling via telephone call motivational interviewing and mailed tailored newsletters about screening and physical activity.  Control: Fruit and vegetable promotion program.  2 groups: Wellness program and control. | Screening uptake.  Colonoscopy, FOBT, sigmoidoscopy, double contrast barium enema, virtual colonoscopy. |  | Screening uptake was higher among participants who remembered receiving a newsletter compared to participants who did not (43.9 vs. 17.7, respectively p=0.02). The newsletter led to higher screening rates among participants who had not screened before. There was no significant difference between intervention (+6.4%) and control group (+4.7%) screening changes at follow-up (p=0.37). |
| Shepherd et al. (31) 2022, Non-randomised experimental study, USA | n=9742 emailed, n=119 completed assessment; Female: 82%; Caucasian (70%); mean age: 52yrs | 3 newsletters mailed or emailed. | Screening uptake. Colonoscopy, FIT. |  | Only 119 (3.9% emailed/mailed) engaged in the study. Of those, 107 ordered a screening kit and 69 (64.5%) participants completed the screening. |

FIT, faecal immunochemical test; FOBT, faecal occult blood test; LCHE, lay community health educator.

**REFERENCES**

1. Peters MDJ, Godfrey C, McInerney P, Munn Z, Tricco AC, Khalil H. Chapter 11: Scoping reviews. 2020. In: JBI Manual for Evidence Synthesis [Internet]. JBI. Accessed March 8, 2022. https://synthesismanual.jbi.global
